# Solution-Processed Functionalized Graphene Film Prepared by Vacuum Filtration for Flexible NO_2_ Sensors

**DOI:** 10.3390/s23041831

**Published:** 2023-02-07

**Authors:** Mbaye Dieng, Siva Sankar, Pingping Ni, Ileana Florea, Pedro Alpuim, Andrea Capasso, Abderrahim Yassar, Fatima Zahra Bouanis

**Affiliations:** 1 COSYS-IMSE, Univ. Gustave Eiffel, 77454 Marne-la-Vallée, France; 2 Laboratory of Physics of Interfaces and Thin Films, UMR 7647 CNRS/Ecole Polytechnique, IP Paris, 91128 Palaiseau, France; 3 International Iberian Nanotechnology Laboratory, 4715-330 Braga, Portugal; 4 Center of Physics, University of Minho, Campus de Gualtar, 4710-057 Braga, Portugal

**Keywords:** graphene nanosheets, liquid-phase exfoliation, functionalization, Co-porphyrin, Fe-Phthalocyanine, nitrogen dioxide, gas sensor

## Abstract

Large-scale production of graphene nanosheets (GNSs) has led to the availability of solution-processable GNSs on the commercial scale. The controlled vacuum filtration method is a scalable process for the preparation of wafer-scale films of GNSs, which can be used for gas sensing applications. Here, we demonstrate the use of this deposition method to produce functional gas sensors, using a chemiresistor structure from GNS solution-based techniques. The GNS suspension was prepared by liquid-phase exfoliation (LPE) and transferred to a polyvinylidene fluoride (PVDF) membrane. The effect of non-covalent functionalization with Co-porphyrin and Fe-phthalocyanines on the sensor properties was studied. The pristine and functionalized GNS films were characterized using different techniques such as Raman spectroscopy, scanning electron microscopy (SEM), transmission electron microscopy (TEM), atomic force microscopy (AFM), X-ray diffraction (XRD), and electrical characterizations. The morphological and spectroscopic analyses both confirm that the molecules (Co-porphyrin and Fe-phthalocyanine) were successfully adsorbed onto the GNSs surface through π-π interactions. The chemiresistive sensor response of functionalized GNSs toward the low concentrations of nitrogen dioxide (NO_2_) (0.5–2 ppm) was studied and compared with those of the film of pristine GNSs. The tests on the sensing performance clearly showed sensitivity to a low concentration of NO_2_ (5 ppm). Furthermore, the chemical modification of GNSs significantly improves NO_2_ sensing performance compared to the pristine GNSs. The sensor response can be modulated by the type of adsorbed molecules. Indeed, Co-Por exhibited negative responsiveness (the response of Co-Por-GNS sensors and pristine GNS devices was 13.1% and 15.6%, respectively, after exposure to 0.5 ppm of NO_2_). Meanwhile, Fe-Phc-GNSs induced the opposite behavior resulting in an increase in the sensor response (the sensitivity was 8.3% and 7.8% of Fe-Phc-GNSs and pristine GNSs, respectively, at 0.5 ppm NO_2_ gas).

## 1. Introduction

Carbon nanomaterials are among the most extensively investigated materials for gas sensing applications [1]. Among this family, graphene nanosheets (GNSs) have been widely used in gas sensor applications, since their discovery in 2004, because their high carrier mobility combined with low intrinsic noise are promising for detection with a high signal-to-noise ratio [2,3,4]. In 2007, Schedin et al. reported the first graphene gas sensor to detect single-molecule gases [3]. They demonstrated that the ultra-high conductivity and the exceptional low noise of graphene originating from its unique two-dimensional (2D) crystal lattice were useful for selectively sensing at room temperature [3]. The mechanism of the detection is based on a charge transfer between GNSs and gas molecules [5]. Solution-processed GNSs offer great potential to fabricate various optoelectronic devices using low-cost techniques [6]. Several methods have been proposed to produce GNSs, including electrochemical exfoliation [7], the reduction of graphene oxide (GO) [8], and the liquid-phase exfoliation (LPE) of bulk graphite in different solvents [9], etc. Hummers’ method to produce GNSs from GO flakes remains the most popular employed method for the exfoliation of GO nanosheets [10]. However, reduced GO flakes contain residual functional oxygen or structural defects that disrupt the sp^2^ carbon bonds between the atoms, which resulted in lower electrical properties [11]. The liquid-phase exfoliation method, i.e., sonication, high-shear mixing, microfluidization, ultrasonication, and ball milling, is a simple strategy to produce 2D nanosheet materials [12]. Sonication is the most common LPE technique employed to exfoliate 2D materials; however, this technique may induce some defects, which is harmful in electrical applications but is beneficial for other applications [13]. Compared to sonication, the shear force technique is best suited to produce a high-yield solution dispersion of 2D materials [14]. This method employs a rotor stator mixer to induce shear forces on the surface of material, which can trigger the large-scale 2D material exfoliation. For instance, this method enables one to produce several hundred liters of 2D material dispersion at one time [15]. A subsequent purification step using a sequential centrifugation is commonly used to obtain 2D materials with a relatively uniform sheet size. This purification procedure is best suited for the fabrication of devices for optoelectronic applications with the consideration of the close relationship between the device performance and sheet sizes of 2D materials.

On the other hand, NO_2_ is one of the major environmental pollutants, which is released from many pathways such as internal combustion engines, fuel combustion, and emissions from industrial complexes [16]. It contributes to the acidity of rain and the degradation of the ozone layer [16]. It was reported that 1 ppm of NO_2_ could cause serious respiratory problems [17]. Real-time monitoring and analysis of NO_2_ gas have proven difficult and constitute a challenging task. As per literature reports, chemiresistive gas sensors have clear attractive advantages over other families of gas sensors because they are easier to integrate with conventional microelectronics technology for implementation into portable devices [18]. Semiconducting metal oxides are the most investigated sensing materials for resistive sensors. However, they suffer from a high working temperature (200–450 °C), a slow response at low concentrations of analytes, poor selectivity, and high power consumption. To resolve these limitations, 2D layered materials, especially carbon nanomaterials (graphene), are a promising alternative; they have the ability to sense gases at room temperature (RT), with good sensitivity, fast response/recovery time, and moderate selectivity. Gas sensors based on graphene and graphene derivatives have already demonstrated the capability of sensing with high sensitivity; however, they suffer from some drawbacks, such as low selectivity and long response and recovery times, which limit their further applications. Various strategies have been employed to combine graphene with other materials to form a new nano-hybrid material with on-demand physicochemical properties and improved sensitive and selective detection of gas molecules. Chemical functionalization via covalent or non-covalent methods remains the most popular approach to fabricate high-performance gas sensors (high response/recovery time, recovery efficiency, high selectivity, and high sensitivity). Several studies demonstrated that graphene is chemically inert and gas molecules are weakly adsorbed, while when it is chemically functionalized, graphene sensors offer better performance for detecting gas molecules due to the presence of the functional group where the analytes can be selectively adsorbed. The adsorbed analyte alters the carrier mobility and density, which in turn results in changes in the conductance of the graphene leading to better sensing properties. Moreover, the functionalization not only improves sensitivity but also may enhance selectivity toward specific gases even at room temperature. A wide variety of functional groups have been used, including polymers [18,19,20], metal oxides [21,22], metal nanoparticles [23], and small organic molecules [24,25]. Metalloporphyrins (MPor) and metallophthalocyanines (MPhc), as functional molecules, have attracted considerable interest due to their planar structure with metal atoms (Co, Fe, Zn, Ni, etc.) at the center and their remarkable physical and chemical properties [26]. They form a self-assembled layer on the surface of carbon nanomaterials via hydrogen bonds, π-π interactions, and electronic repulsion [27]. Their ability to chelate different elements of the periodic table to form a large variety of organo-metallic compounds with novel functionality is beneficial for sensing applications. Regarding the non-covalent functionalization of graphene, MPor and MPhc exhibit strong dipole interactions with analytes that can alter the main charge carrier concentration and consequently modulate its conductivity [28,29,30]. For instance, Zhou et al. [31] reported that a hybrid material composed of reduced GO and copper-phthalocyanine exhibited excellent response, reversibility, and selectivity toward NH_3_ compared to pristine reduced GO. Mackin et al. [32] demonstrated that functionalized graphene with cobalt porphyrin exhibits excellent selectivity over interfering compounds such as water and common organic solvents. Guo et al. [33] showed that functionalized graphene with porphyrin presents greater detection toward dinitrotoluene, 2,4,6-trinitrotoluene, 1,3,5-trinitrobenzene, and 1,3-dinitrobenzene and good reproducibility compared with pristine graphene. Ikuta et al. [34] demonstrated that selectivity toward NO_2_ can be achieved by using metalloporphyrin molecules [34].

In this work, we report a simple and easy-to-fabricate chemiresistive sensor based on functionalized solution-processed GNSs, which are used to detect selectively NO_2_ molecules. GNSs were produced through the high-shear exfoliation of graphite in an aqueous solution. We demonstrated the formation of GNSs by X-ray diffraction, Raman spectroscopy, and high-resolution transmission electron microscopy. The produced GNSs are of high quality and mostly less than a few layers thick. This method produces a stable ink of exfoliated GNSs, on a large scale at a relatively low cost, for use in printed gas sensors. Free-standing GNS films fabricated by vacuum-assisted filtration were used to fabricate NO_2_ sensors. The vacuum filtration method offers a simplified process to manufacture a thin-film network of GNSs, with a porous structure and a high specific surface area, which are of great importance for gas sensing. The selectivity issue is addressed by functionalizing the surface of GNSs with MPor and MPhc, as we have recently demonstrated [35]. Our findings show the utility of the LPE method to fabricate flexible gas sensors and the potential of the non-covalent functionalization of GNSs for gas sensing applications. Moreover, we used the LPE method to produce dispersions of GNSs, which, according to our best knowledge, has not been investigated before to fabricate NO_2_ gas sensors.

## 2. Materials and Methods

### 2.1. Preparation of Graphene

Graphene dispersion was prepared by a simple, eco-friendly, and low-cost LPE. In this method, a green solvent cyrene was used as a liquid medium, and shear force was used to exfoliate the natural graphite (NG) into GNs in cyrene. A total of 20 g of NG powder of lateral size (600–800 µm, From Merck-Portugal) and 1 v% of disperbyk-2012 (BYK-Wesel-Deutschland) was added to 500 mL of cyrene. Thirty minutes of ultrasound was used to obtain the proper mix of the solution. The prepared solution was allowed to exfoliate using a high-shear mixer (Silverson L5M (Silverson-UK), standard mixing with an axial flow head) at 6000 rpm for 6 h at room temperature. An ice bath was used during the entire process to dissipate the heat that was being generated during the exfoliation process. After this, the suspended black-color exfoliated mixture was collected and centrifuged at 6000 rpm for 30 min to remove thick and un-exfoliated flakes, and the supernatant containing GNs was separated. The concentration of the GNs was measured by a simple thermogravimetric analysis, and it was estimated to be 1.4 mg/mL.

### 2.2. Functionalization of Graphene Nanosheets

5,10,15,20-Tetraphenyl-21H,23H-porphine cobalt(II) (Co-Por) (85%) and iron (II) phthalocyanine (Fe-Phc) (90%), as well as organic solvents (dichloromethane (DCM) and acetone), were purchased from “Merck-France” and used without further purification. **Preparation of nanohybrid materials:** The functionalization of the graphene was achieved following our previously published procedures [35]. The Por or Phc compounds were dissolved in DCM at a 10^−4^ mol/L concentration for Co-Por, or a 5 × 10^−4^ mol/L Fe-Phc concentration. A total of 10 µL of Por or Phc solution was drop-cast onto the surface of a GNS. Then, the sample was dried for one hour at 100 °C and then washed several times with acetone to remove the unadsorbed molecules. The functionalized graphene (Co-Por-graphene or Fe-Phc-graphene) was then dried at 40 °C for 5 min.

### 2.3. Characterization Methods

**Scanning electron microscopy** (SEM) was performed using a Hitachi S4800 microscope. Chemical state of functionalized graphene was analyzed using **energy dispersion spectroscopy** (EDS) (Thermo Ultra Dry-Electron corporation-USA). **Raman spectra** were obtained using a high-resolution confocal Raman microscope (Labram HR800; HORIBA Jobin Yvon, Palaiseau-France) with a microscope lens of 100 (NA = 1). Micro-Man mapping was performed in high-resolution mode, using a laser excitation of λ = 532 nm with 20 s scan time and two accumulations per spectrum. The number of arrays in the Raman spectrometer was 600 grooves per mm. Raman mapping was performed on 5 μm × 5 μm zones with a pitch of 0.2 μm. **Transmission electron microscopy (TEM)** analyses were performed using a Titan-Themis Thermo Fisher electron microscope operating at 80 and 300 kV. To probe the presence of components of the porphyrin (Co) or phthalocyanine components (Fe), energy dispersion spectroscopy (EDS) analyses were performed on a Titan-Themis 200 kV (ThermoFisher Scientific-Netherlands). **X-ray diffraction measurements** were acquired on a Bruker D8 Discover (Deutschland) diffractometer using a Cu Kα source (λ_1_ = 1.5406 A, λ_2_ = 1.5444 A), and 2θ scans were obtained from 5 to 80°. The spacing between graphene sheets was determined using Bragg’s law: d = λ/(2 sin θ) where 2θ refers to the position of the maximum intensity of the GNS diffraction peak in the samples. **Atomic force microscope (AFM)** images were acquired using a Dimension Icon instrument from Bruker with Scan A system which operated in tapping mode using standard AFM probe featuring pyramidal silicon tip (325 kHz, 40 N/m).

**Device fabrication:**

The electrodes were deposited directly on the surface of the GNSs film using silver paste (Figure S1). The gap between the two electrodes was 5 mm.

**Electrical measurements:**

The electrical measurements were performed using a semiconductor parametric Keithley 4200-SCS (Tektronix company –Les Ulis-France) analyzer under ambient conditions. The measurements were carried out by applying a fixed-bias voltage of 500 mV between the two electrodes.

**Sensing measurement:**

The sensor tests were carried out by using an Owlstone gas generator (V-OVG) (Westport, USA) based on permeation tube technology (as illustrated in Figure S2). This system enables one to maintain precise and repeatable concentrations of chemicals and calibration gas over long periods. NO_2_ permeation rate of 2425 ng/min at 30 °C was used. Dry N_2_ (99.99%) was used as the dilute gas in order to reach the desired concentration. The gas generator is connected to Nextron chamber with micro probe system analysis. This system enables in situ measurement of the electrical properties of devices under various environmental conditions (vacuum, temperature, gas flow, humidity, light irradiation, etc.). The chamber is connected to precision humidity control system (1–97% relative humidity (RH) and 0.01 resolution) and temperature control system (20–200 °C). Prior to measurements, the chamber was purged with N_2_ flow until the resistance of sensor was stabilized. Then, the sample was annealed at 100 °C in order to desorb all volatile molecules and residues on the surface of GNSs. The response of sensor upon exposure to NO_2_ is defined as:

(1) $$S_{\mathrm{resp}}\;(\%)=\frac{R-R_{0}}{R_{0}}\;\times \;100$$

where R_0_ is the resistance of the sensors before exposure to NO_2_, and R is the resistance in the presence of NO_2_ gas. The response time of the sensor is defined as the time needed to reach 90% of the final resistance. Recovery time is defined as the time required for a sensor to return to 90% of the original baseline signal upon desorption of NO_2_. The reference response of the device was measured under dry N_2_ atmosphere. All gas-sensing tests were carried out under room temperature 30 ± 2 °C, and the relative humidity was 30%. During gas exposure, the electrical measurements were performed using a semiconductor parametric analyzer Keithley 4200-SCS. After exposure time of the device under NO_2_/N_2_ of 5min, the device was exposed to a constant flow of pure N_2_ for 15 min at 100 °C in order to desorb the NO_2_ molecules adsorbed on the device.

## 3. Results and Discussion

### 3.1. Raman Spectroscopy

**Figure 1:**
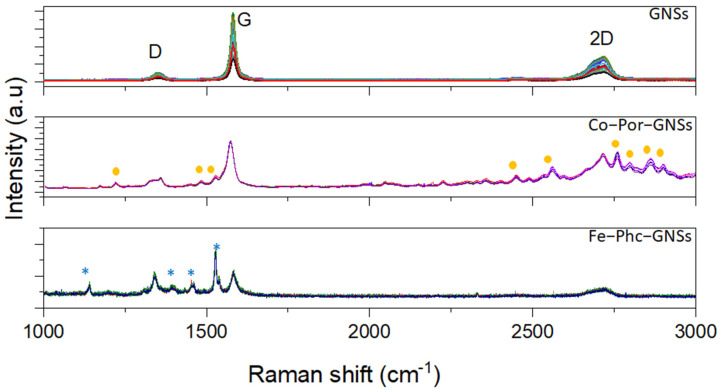
Raman spectra of (top to bottom) solution-processed graphene nanosheets (GNSs), Co-Por-GNSs, and Fe-Phc-GNSs (λexc = 532 nm).

Micro-Raman spectroscopy was used to characterize the defect densities of GNSs and functionalized GNSs. Further, Raman spectroscopy is a powerful tool to use to investigate electron transfer interactions between the GNSs and MPor and MPhc. All Raman spectra were recorded at the excitation wavelength of 532 nm under ambient conditions (laser spot size is 0.2 µm on the sample). The data were obtained using minimum power in order to prevent laser heating. Both GNSs and functionalized GNSs showed three typical peaks assigned as D, G, and 2D peaks observed at around 1353, 1581, and 2716 cm^−1^, respectively (Figure 1), which are consistent with previous studies [36]. The D band at 1353 cm^−1^ originates from structural defects, which may arise from certain defects such as vacancies, grain boundaries, and amorphous carbon species [37]. The G band at around 1581 cm^−1^ is attributed to the first-order diffusion of the E2g mode, and the 2D band at 2716 cm^−1^ is the most important band confirming the presence of graphene in the structure and attributed to the two-phonon double resonance process indicator of the number of graphene layers [38]. The fitting 2D band enables one to determine the number of layers [36]. Zhou et al. [39] attributed the peaks at ~2707 cm^−1^ and ~2732 cm^−1^ to a few and multilayer graphene sheets. In addition, the 2D band is broadened which may be due to the fact that the shear-assisted LPE GNSs contain a few layers with some defects. Moreover, the observed weak D band indicates high-quality graphene with the presence of few defects in the crystalline structure. In the Raman spectra of functionalized GNSs (Co-Por-GNS and Fe-Phc-GNSs), new peaks appear which are assigned to the physi-adsorbed molecules. The intensity ratio of the D and G peaks *(I(D)/I(G)),* usually used to provide quantitative metrics of the defect densities of graphitic materials, was found to be 0.21, 0.23, and 0.19 for pristine GNSs, Co-Por-GNSs, and Fe-Phc-GNSs, respectively. The *I(D)/I(G)* defect ratio found for pristine GNSs (0.21) is much lower than the defect ratio of 0.43 previously reported using the sonication method [40]. As shown in Figure 1, the D and G peak intensities were almost unchanged after functionalization, thus confirming that the functionalization does not destroy the extended π-conjugation system of the GNSs [41]. The position of the G band is sensitive to the functionalization of GNSs with Co-Por and Fe-Phc. Indeed, the G band of Co-Por-GNSs appears at 1573 cm^−1^ which is downshifted by 8 cm^−1^ compared to that of GNSs at 1581 cm^−1^, a clear indication of molecular doping with Co-Por molecules, which induce n-type doping, e.g., an increasing electron concentration in pristine GNSs. On the contrary, the G band of Fe-Phc-GNSs observed at 1583 cm^−1^ is upshifted which suggests p-type doping caused by Fe-Phc molecules, e.g., a decreasing electron concentration in the GNSs. These observations are in agreement with our previous report on interfacial charge transfer interactions, via π-π interactions, between CVD-graphene and Fe-Phc and Co-Por [35].

### 3.2. X-ray Diffraction Analysis

**Figure 2:**
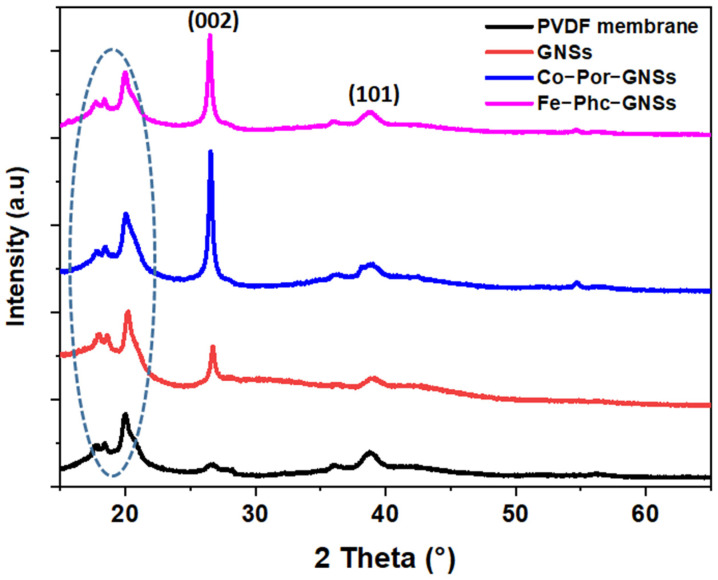
XRD patterns (bottom to top) of GNSs, Co-Por-GNSs, Fe-Phc-GNSs, and PVDF membrane. The marked circle are characteristic of PVDF membrane peaks.

X-ray diffraction (XRD) was utilized to confirm the crystallinity of the shear-assisted LPE of GNSs. The XRD patterns of GNSs and functionalized GNSs with Co-Por and Fe-Phc recorded in the range of 2θ from 15° to 65° are presented in Figure 2. The XRD patterns show the presence of a strong sharp diffraction peak located at 2θ = 26.6° (002) with an interlayer distance of 0.33 nm, determined using Bragg’s law equation [42,43]. Besides this peak, one peak appeared at 2θ = 39° and was assigned to a short-range order in stacked graphene layers (101) [42,43]. The polyvinylidene fluoride (PVDF) membrane has peaks at around 2θ angles of 18.4 and 20.2 at planes (100) and (021) (marked by circle in Figure 2). After functionalization, no effect was observed on the XRD patterns which allows us to conclude that neither Co-Por nor Fe-Phc affect the crystalline structure of GNSs [44].

### 3.3. Scanning Electron Microscopy (SEM) and Energy-Dispersive Spectroscopy (EDS)

**Figure 3:**
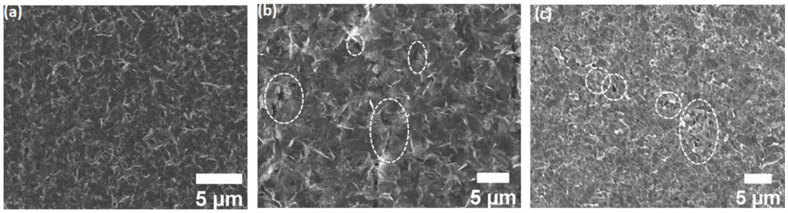
SEM images of (**a**) pristine GNSs, (**b**) functionalized GNSs with Co-Por, and (**c**) functionalized GNSs with Fe-Phc. The observed aggregates on functionalized surface are marked by circles.

SEM observations were performed in order to obtain more information on the morphology of GNSs, Co-Por, and Fe-Phc functionalized nanosheet networks as well as the homogeneity of the functionalization. SEM images of graphene nanosheet networks are shown in Figure 3 and Figure S3. The low-magnification image shows that nanosheets form a uniform pinhole-free film of an interconnected nanosheet network with apparently different levels of thickness, covering the whole substrate (Figure 3a and Figure S3a). The thickness of the nanosheet network is measured to be 1.5 µm. Figure S1 also confirms no variation in the cross-sectional morphology of the nanosheet film. Once it is functionalized with Co-Por and Fe-Phc, one can see the presence of molecular aggregates with different sizes (marked by circles in Figure 3b,c) on the surface of the graphene sheets (Figure 3b,c and Figure S3), which originates from the adsorption of the molecules. To gain more insights into the chemical composition of these aggregates, we performed energy-dispersive spectroscopy (EDS). The spectra of pristine GNSs, Co-Por, and Fe-Phc functionalized GNSs are shown in Figure S4 and the inset of Figure S4. The EDS measurements of pristine GNSs display signals of C (elements of graphene), Si, and O (elements of the PVDF membrane), while samples functionalized with Co-Por and Fe-Phc additionally display N and Co and N and Fe, respectively (Figure S4). These results suggest that the Co-Por and Fe-Phc are physi-adsorbed onto the surface of GNSs.

### 3.4. Transmission Electron Microscopy (TEM) and Energy-Dispersive Spectroscopy (EDS)

**Figure 4:**
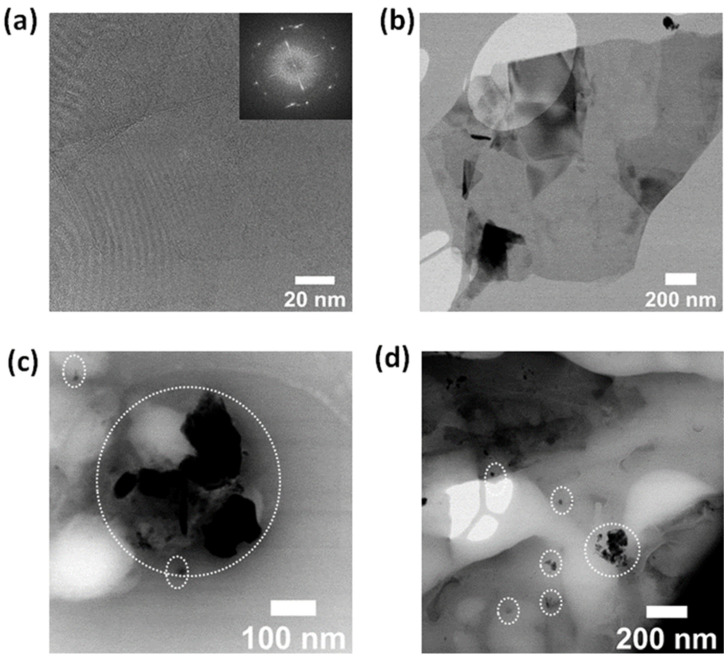
High-resolution transmission electron microscopy (HRTEM) images of the GNSs transferred onto TEM grids before (**a**,**b**) and after functionalization with Co-Por (**c**) and Fe-Phc (**d**). FFT image (in the Figure 4a inset).

Extensive transmission electron microscopy (TEM) characterizations were performed to obtain more information on the morphological and chemical characteristics of pristine and functionalized nanosheets. Figure 4 displays high-resolution TEM (HRTEM) images of pristine and functionalized GNSs, which were scratched off from the substrate, and their corresponding fast Fourier transform images (FFT; see Figure 4a inset). The TEM analysis of pristine GNSs reveals that the majority of the exfoliated GNSs are characterized by lateral dimensions between one and five microns (Figure S5). A selected area is seen in the FFT image (Figure 4a inset and Figure S5) indicating the highly crystalline structure of the GNSs. The HRTEM images (Figure 4a,b and Figure S5) taken of a large area confirm the uniform surface over a large area, and the cross-sectional view reveals the high crystallinity of graphene flakes. The number of graphene layers was estimated by analyzing the edge structure of the nanosheets, where few-layer structures were observed, which is in good agreement with the Raman results discussed above. Meanwhile, morphological investigations of Co-Por-GNSs and Fe-Phc-GNSs show that the molecules aggregate and form islands with different sizes (see Figure 4c,d and Figure S6b,c) with the simultaneous presence of small aggregates (1–5 nm) and big aggregates on the surface of graphene (10–50 nm). These structural observations were completed by chemical analyses performed on different areas of the sample using the EDS technique in the scanning transmission electron microscopy (STEM)-high-angle annular dark field (STEM-HAADF-EDS) imaging mode of the electron microscope (Figure S7 in SI). This analysis enables better identification of the presence of the Co-Por and Fe-Phc components on top of the graphene nanosheet structure. As elements of interest, we chose the K edges of Carbon (C) at 0.282 keV, Nitrogen (N) at 0.392 keV, Iron (Fe) at 6.4 keV, and Cobalt (Co) at 0.78 keV. In Figure S7, we can identify the elemental signals of Co and Fe, which suggests the presence of molecules Co-Por and Fe-Phc on the top surface of the GNS.

**Figure 5:**
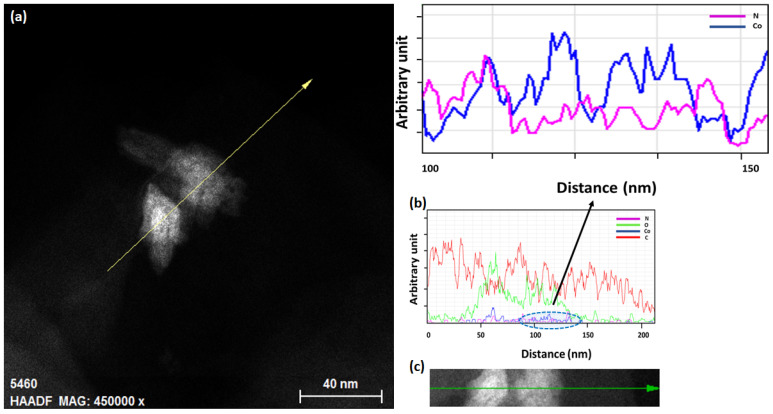
STEM-HAADF-EDS line scan analyses of Co-Por functionalized GNSs: (**a**) STEM-HAADF micrograph on the chosen area where a line scan along the direction indicated by the yellow arrow was performed. (**b**) Corresponding STEM-EDS line scan spectrum showing the variation of the Nitrogen (in pink), Cobalt (in blue), Carbon (in red), and oxygen (in green) signal recorded along the scanning direction indicated by the green arrow in (**c**). (**c**) Zoom of the analyzed area indicated by the yellow arrow in (**a**).

Figure 5a–c shows STEM-EDS line scan analyses recorded on a chosen area on the surface of Co-Por-GNSs. The analyses performed on Fe-Por-GNSs are illustrated in Figure S7 in SI. In Figure 5a–c, we can identify the elemental signal of C that originates from GNSs. Additionally, we observe the elemental signal of Co and N indicating the presence of Co-Por on the surface of GNSs. Similar results were obtained for Fe-Phc functionalized GNSs (see Figure S8).

### 3.5. Atomic Force Microscopy (AFM)

**Figure 6:**
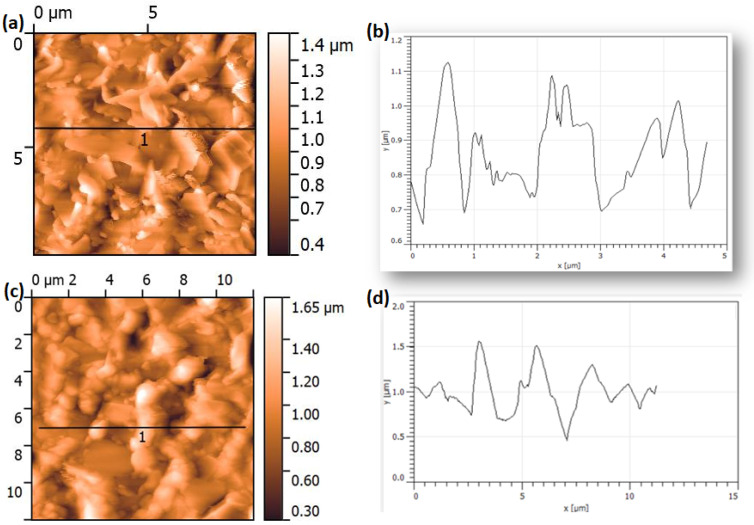
AFM image of GNSs (**a**) and Co-Por-GNSs (**c**). High profile of the black line in the image of GNSs (**b**) and Co-Por-GNSs (**d**).

The morphology of pristine GNSs was further investigated by AFM; the results are shown in Figure 6 and Figure S9. The AFM images show a clear stacked structure composed of connected GNSs. The height profile of the AFM image (Figure 6a) indicated that the surface roughness of the nanosheet network thin film is estimated to be around 0.1–1.5 µm, suggesting the presence of multilayer graphene. AFM morphology and surface roughness do not change much upon functionalization (Figure 6c,d); only the height was slightly increased.

### 3.6. Investigation of Sensing Capabilities of Pristine and Functionalized GNSs

**Figure 7:**
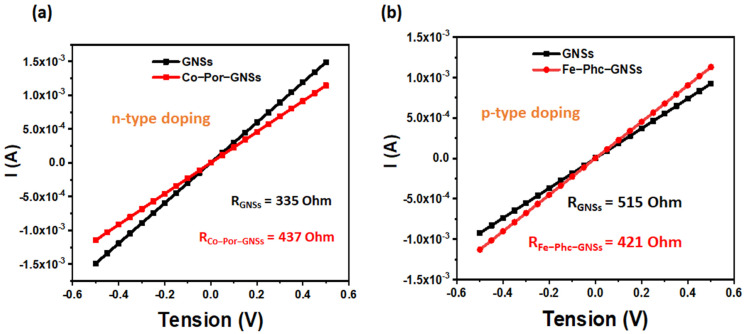
I versus V curves of GNSs and functionalized GNSs with Co-Por and Fe-Phc: (**a**) GNSs and Co-Por-GNSs; (**b**) GNSs and Fe-Phc-GNSs.

Before investigating the sensing behavior of pristine and functionalized GNSs, we describe, in this section, the detailed electrical measurements performed using a two-point probe measurement with silver paste as ohmic contacts. Figure 7a,b show the current versus voltage for devices based on pristine GNSs, Co-Por-GNSs, and Fe-Phc-GNSs. The measurements were recorded with an applied sample bias of 500 mV (see Figure S10). As shown in Figure 7, the (I–V) curves exhibit a linear ohmic behavior, suggesting an ohmic-type sensor rather than a Schottky-based sensor, which is perfect for fabricating sensing devices. Before gas sensing experiments, the resistance of pristine and functionalized GNSs was investigated. The resistance of pristine GNS networks is about 335 Ω/sq (Ω/sq), which is slightly increased to 437 Ω/sq after functionalization with Co-Por. However, after functionalization with Fe-Phc, a significant difference in the resistance is exhibited. The difference in resistance is now a factor of ~18% lower than that of pristine GNSs (515 Ω/sq for GNSs and 421 Ω/sq for Fe-Phc-GNSs). This behavior was observed consistently in various devices prepared under similar conditions (see Figure S11 in supporting information). The functionalized samples’ resistance change is attributed to the doping effect on the GNS surface upon the adsorption of the molecules. These observations agree with our previous work [35], where we demonstrated that the Co-Por induces n-type doping of graphene, suggesting an electron transfer from Co-Por to graphene, while Fe-Phc causes p-type doping. The statistical results confirm this behavior (Figure S11 in supporting information), and the observed shift is compatible with the doping-induced change in Fermi energy and arguments for a charge transfer between the GNSs and molecules. All these observations agree with Raman experiments, which confirm that the Co-Por causes n-doping while Fe-Phc results in the p-doping of graphene.

Nitrogen dioxide (NO_2_) toxic gas, emitted primarily from fossil fuel combustion, automobiles, and industry, is harmful to human health and the environment. Therefore, there is a growing need for developing gas sensors operating at room temperature and detecting NO_2_ gas both qualitatively and quantitatively [16,17,45]. To demonstrate the utility of these functionalized GNSs as chemiresistive sensors, both pristine and functionalized thin-film networks of GNSs were exposed to NO_2_ gas at room temperature (30 ± r is composed of a 100 cm^3^ box made of stainless steel and equipped with a precision humidity control system (1–97% RH and 0.01 resolution) and a temperature control system (20–200 °C) attached from outside to the chamber. As previously demonstrated, heating accelerates the desorption process and quick recovery of the sensor, through vibrationally exciting molecules and then repulsing them from the surface [16]. A known volume of NO_2_ is injected into the chamber and then diluted with dry N_2_ to fill up the volume of the chamber. The NO_2_ vapor concentration is calculated by using the following formula:

(2) $$q_{d}=\frac{CxQ}{\frac{22.4}{M}}$$

where q_d_ is the permeation rate (ng/min), C is the concentration in ppm, Q is the flow rate (ml/min), and M is the molecular weight of gas (g/mol). When exposed to NO_2_, the change in sheet resistance response is recorded for pristine and functionalized GNS films.

**Figure 8:**
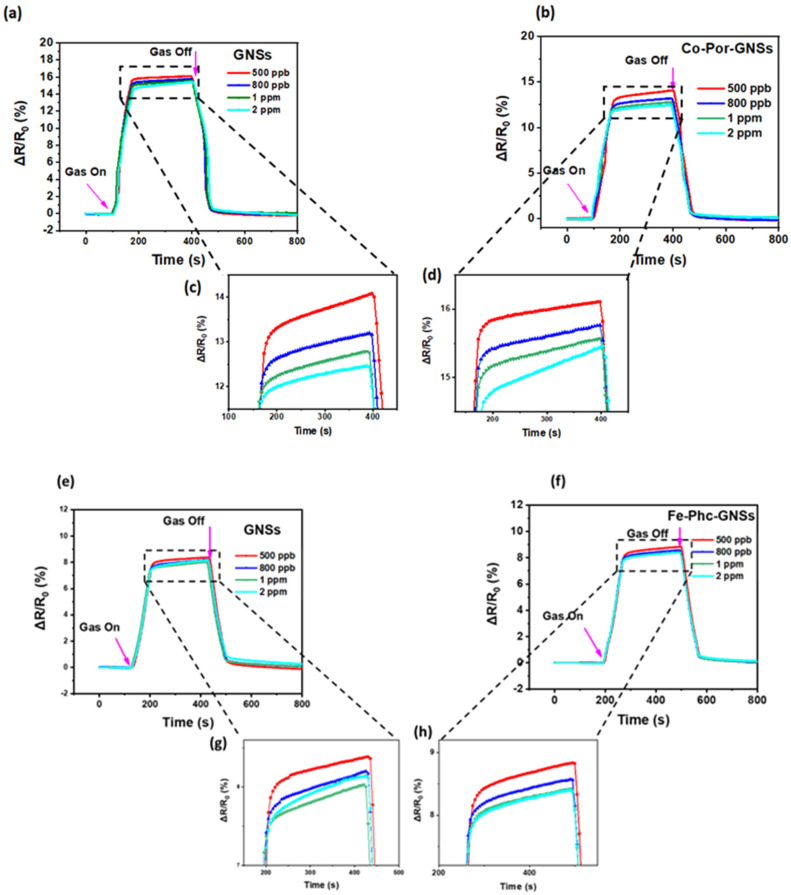

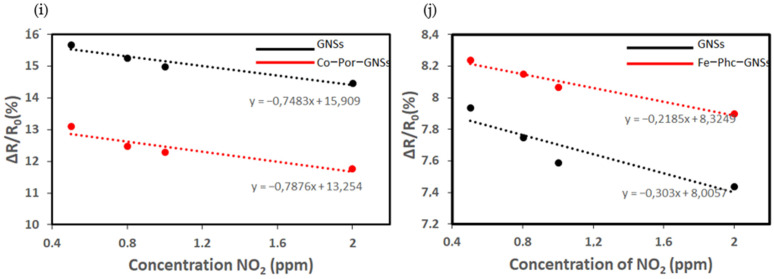
(**a**–**j**)The response of pristine GNS sensor and functionalized GNS sensors to NO_2_ gas under various concentrations ranging from 0.5 ppm to 2 ppm.

Figure 8a,c show typical responses of the GNS sensor toward various concentrations of NO_2_ (0.5–2 ppm) recorded at room temperature. The values of the response and recovery time determined upon exposure of the GNS thin film to 0.5 ppm of NO_2_ are listed in Table 1. The exposure of the GNS thin film to NO_2_ led to a change in the sensor resistance (Figure 8 and Figure S12a). For measuring the response, a fixed bias of 500 mV was applied. The results reveal that the GNS sensor lightly responds to various concentrations of NO_2_. For a low concentration of (NO_2_) = 0.5 ppm, the response of the pristine GNS device is 15.69%, while it is 14.48% for 2 ppm, indicating a low responsivity of the devices. This result shows that the GNS film responds to a low concentration of NO_2_ (Figure 8 and Figure S12a). This response is assumed to be related to a charge transfer induced by NO_2_ molecule adsorption on graphene acting as an acceptor [46]. The GNSs are initially covered with residual water molecules originating from the preparation process, which induce p-doping. When NO_2_ molecules are adsorbed on the GNS surface, they act as electron acceptors. Therefore, they attract electrons from p-doped graphene, hence the shift in the GNSs’ Fermi level [47] leading to an increase in hole concentration, which is consistent with an increase in the conductance, e.g., a decrease in the resistance of the GNS film as the NO_2_ concentration increases [3,5].

**Figure 9:**
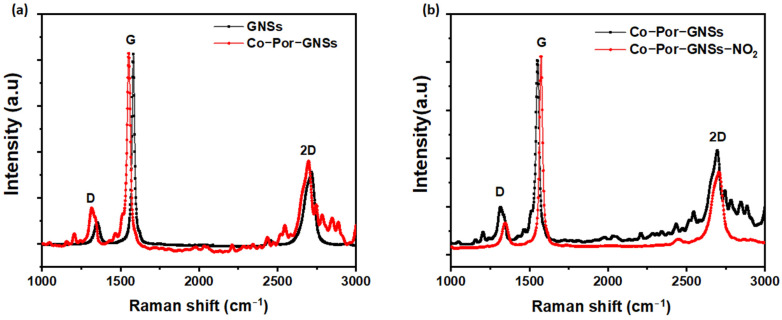
Raman spectra of GNSs and Co-Por-GNSs before (**a**) and after (**b**) detection of NO_2_ (λexc = 532 nm).

To study the effect of functionalization on the GNS sensor performance, the response of functionalized GNS thin films to different NO_2_ concentrations was studied in similar conditions (Figure 8b,d and Figure S12). In the case of the Co-Por-GNS sensor, a significant decrease in the sensor response was observed as the concentration of NO_2_ increased, compared with pristine GNSs (Figure 8b,e and Figure S12). For example, the response of the functionalized device Co-Por-GNSs is 13.10% after exposure to 0.5 ppm of NO_2_, while the value is only 11.84% for 2 ppm. These observations are consistent with a previous study by Yuan et al. [48]. They demonstrated that the chemical modification of reduced GO with sulfonic acid or amine groups significantly improves the sensing performances of NO_2_ sensors. However, the responsiveness of the Fe-Phc-GNS sensor exhibited the opposite behavior resulting in an increase in responsiveness in the positive direction (Figure 8d). These two different behaviors were attributed to different electrical conduction pathways of the sensors. When Co-Por molecules are adsorbed on GNSs, they induce n-type doping, which increases electron concentration, decreasing the electrical resistance of GNSs. The high response of Co-Por to NO_2_ may be attributed to the coordination of NO_2_ with Co atoms or a redox reaction with the porphyrin ring of Co-porphyrin [49,50]. To better understand the response of the functionalized GNSs to NO_2_, Raman spectra were recorded before and after the adsorption of gas molecules. The Raman spectra recorded after the NO_2_ adsorption process showed a significant blue shift in the position of G and 2D bands (Figure 9), indicating GNS p-doping, which makes GNSs more resistive since Co-Por-GNSs were already n-doped. These results suggest that Co-Por molecules assist in the capture of NO_2_ gas via a redox reaction.

On the other hand, positive responsiveness was observed when Fe-Phc-GNS films were tested, e.g., the responses of Fe-Phc-GNSs after exposure to 0.5 ppm and 2 ppm were 8.3% and 7.7%, respectively, while the responses of pristine GNSs under the same concentrations were 7.8% and 7.2%, respectively (Figure 8c,d). In this case, the electrons are transferred from GNSs to Fe-Phc, resulting in the p-doping of GNSs. Upon exposure to NO_2_, an electron transfer from Fe-Phc to NO_2_ leads to the compensation of p-type charge carriers in the GNSs, resulting in increased sensor resistance.

Figure 8e,f plot the response (or sensitivity) of the two sensors, pristine and functionalized devices, at different NO_2_ concentrations. These plots show excellent linear correlation, which is crucial for practical device applications, and the functionalization improves the sensitivity of GNSs at low concentrations of NO_2_ gas.

The performance of our sensors was compared with the available research articles and is presented in Table 2. It is clear that the Co-Por-GNS and Fe-Phc-GNS sensors show better sensing performance for their response/recovery times compared to other reported graphene-based sensors, which can be further improved by the optimization of the sensor structure and by using a thinner film of GNSs.

**Figure 10:**
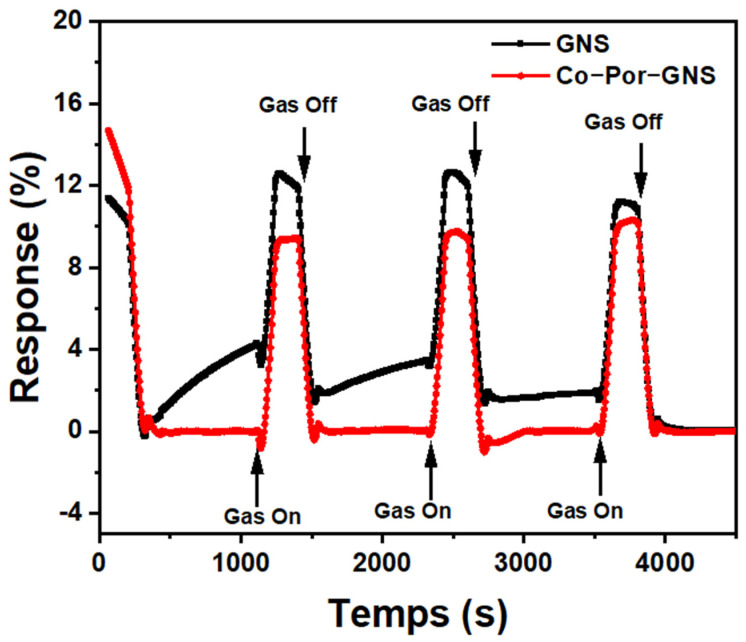
The repeatability properties of the GNSs and functionalized GNSs with Co-Por sensor exposed to 1 ppm NO_2_.

Figure 10 shows a real-time response of the two devices Co-Por-GNSs and GNSs when subjected to various absorption/desorption cycles of NO_2_ at 1 ppm concentration to evaluate repeatability. As evidenced by this figure, the functionalized GNS-based gas sensor exhibits a high-reproducibility characteristic during a long time in this condition without any significant drift after turning off the NO_2_ and purging the chamber with N_2_. Thus, the functionalized GNS sensor was shown to possess good repeatability and reversibility, demonstrating relatively fast and stable on/off switching in each cycle. Furthermore, even after several months, the functionalized GNS sensors still show excellent sensing performance, which suggests that these sensors have great potential in the sensing area.

**Sensing mechanism:** The possible gas sensing mechanism of functionalized GNSs is as follows.

**Figure 11:**
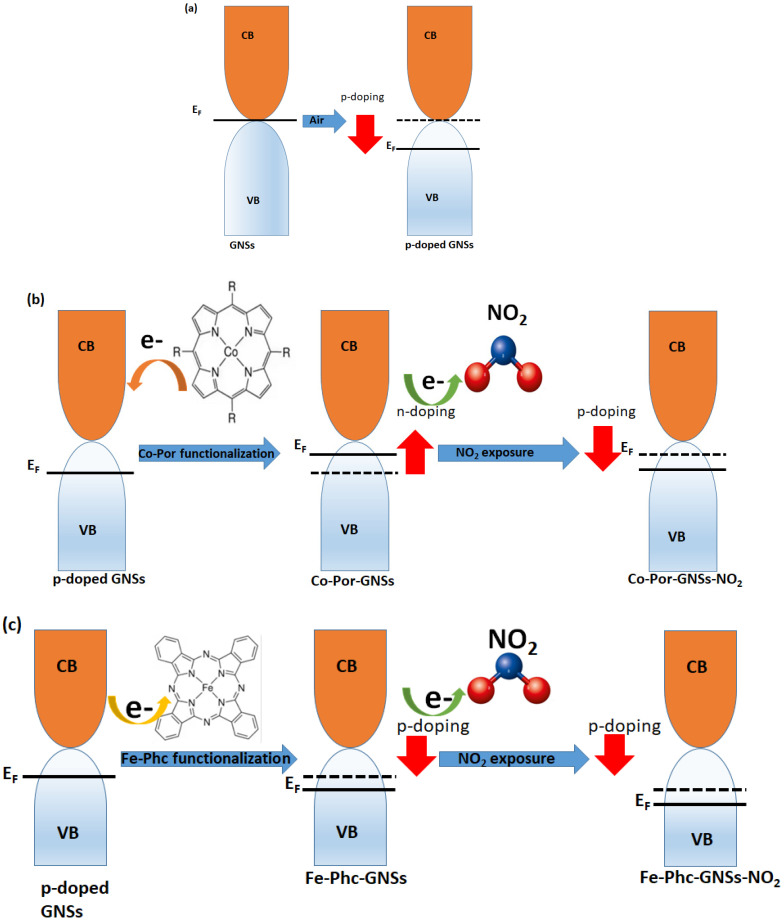
Schematic band diagram of the GNSs and functionalized GNSs before and after exposure to NO_2_.

In ambient conditions, the GNS is p-doped due to stabilized anions (O_2_^−^) available on its surface. They will receive electrons from the GNS valence band [47], which induce its hole doping (Figure 11a). In our previous work, we reported that graphene is p-doped by functionalization with Fe-Phc and n-type doped with Co-Por [35]. Indeed, the chemical modification of GNSs with Co-Por causes a charge transfer between Co atom d-electrons and GNSs’ π-electrons; as a result, there is an increase in the electron concentration (n-type doping), and the Fermi level shifts toward the conductance band (Figure 11b). When NO_2_ molecules are adsorbed on the Co-GNS surface, they act as electron acceptors (the adsorption of NO_2_ on n-doped graphene can be facilitated between electron-deficient NO_2_ and electron-rich graphene). Therefore, they extract electrons from Co-Por-GNSs, hence the shift in the GNSs’ Fermi level toward the valence band (Figure 11b). Meanwhile, the adsorption of Fe-Phc on the GNS surface induces a charge transfer from GNSs to Fe-Phc thus indicating p-type doping. Upon exposure to NO_2_ molecules, the latter are adsorbed on the Fe-Phc-GNS surface, and an electron transfer from Fe-Phc to NO_2_ occurs resulting in an increase in hole density, and as a result, the Fermi level shifts more toward the valance band (Figure 11c).

## 4. Conclusions

In summary, an easy and cost-effective method to produce NO_2_ sensors using solution-processed GNSs followed by non-covalent functionalization with Co-Por and Fe-Phc on a PVDF membrane was demonstrated. The investigation of the surface morphology before and after functionalization showed the presence of physi-adsorbed molecules on the GNS surface. Raman spectroscopy and electrical studies demonstrated an effective charge transfer between GNSs and functionalized molecules confirming the non-covalent π-π stacking of aromatic molecules on GNSs. The functionalized sensor was then tested under various concentrations of NO_2_ to highlight the effect of the functionalization. The results showed the responsivity of sensors to a low concentration of NO_2_ (5 ppm). Moreover, upon exposure to NO_2_ molecules, the functionalized GNSs showed improved responsiveness compared to the pristine GNSs. We demonstrated that the type of functionalized molecules was found to be an important factor influencing the GNS sensor response: Fe-Phc exhibited positive responsiveness while Co-Por resulted in a decrease in responsiveness due to the charge transfer caused by the molecule. This novel GNS-based gas sensor displayed good reproducibility. These results indicate that the combination of GNSs with Por and Phc is a promising approach to develop performed sensors for application in environmental monitoring.

## Figures and Tables

**Table 1:** Responsivity and recovery time of pristine GNS and functionalized GNS gas sensors exposed to 0.5 ppm of NO_2_ at room temperature.

Sample	Response time (s)	Recovery time (s)
GNSs	72	70
Co-Por-GNSs	63	54
Fe-Phc-GNSs	63	63

**Table 2:** Sensing properties comparison with literature.

Material Used	Type of Sensors	Operating Temperature	Detection Range	Sensitivity	LOD (ppb)	Res/Rec (Time) (s)	Ref.
Mo-Porphyrin modified graphene	FETs	RT	2–800 ppb	Not reported	0.3	Not reported	[34]
Eu(TPyP)(PC)/rGO	Chemiresistive	RT	0.5–100 ppm	Not reported	80	172/828 (20 ppm)	[51]
Polymer/rGO	Chemiresistive	RT	0.15–5 ppm	1.03 ppm^−1^	150	180/360 (0.5 ppm)	[52]
BNG	Chemiresistive	RT	1–80 ppb	0.05%	Not measured	177/392 (1 ppb)	[53]
Sulphur doped graphene	MEMS	RT	500 ppt–100 ppm	1.8%	0.5	-	[54]
Co-Por-GNSs	Chemiresistive	RT	0.5–2 ppm	13.1%	Not measured	63/54 (0.5 ppm)	This work
Fe-Phc-GNSs	Chemiresistive	RT	0.5–2 ppm	8.3%	Not measured	63/63 (0.5 ppm)	This work

## Data Availability

Not applicable.

## Supplementary Materials

The following supporting information can be downloaded at https://www.mdpi.com/article/10.3390/s23041831/s1. Figure S1: Schematic view of (a) measurement setup for resistance, (b) GNS sample with silver paste, Figure S2: Schematic view of setup used for gas sensing measurement, Figure S3: Scanning electron microscopy (SEM) images of (a) GNSs prepared by liquid-phase exfoliation and filtered on PVDF membrane, (b) GNSs functionalized with Co-Por, and (c) GNSs functionalized with Fe-Phc, Figure S4: Energy-dispersive X-ray analysis (EDS) spectra of GNSs (a) and functionalized GNSs with Co-Por (b) and Fe-Phc (c), Figure S5: TEM image of GNSs obtained from different areas and its corresponding electron diffraction pattern in inset, Figure S6: HRTEM and STEM-BF micrographs showing GNS film (a) obtained from different areas on the surface and functionalized GNSs with Co-Por (b) and Fe-Phc (c), Figure S7: STEM-HAADF-EDS chemical mapping recorded on a larger area of GNSs (a) and functionalized GNSs with Co-Por (b) and Fe-Phc (c) giving an indication of the elemental percentage on each analyzed area, Figure S8: STEM-HAADF-EDS line scan analyses of Fe-Phc functionalized GNSs: (a) STEM-HAADF micrograph on the chosen area where a line scan along the direction indicated by the yellow arrow was performed. (b) Corresponding STEM-EDS line scan profile showing the variation of the Nitrogen (in pink), Iron (in blue), Carbon (in red), and oxygen (in green) signal recorded along the scanning direction indicated by the green arrow in (c). (c) Zoom of the analyzed area indicated by the yellow arrow in (a), Figure S9: AFM image of GNSs (a), Co-Por-GNSs (c), and Fe-Phc-GNSs (e). Height profile of the black line in image of GNSs (b), Co-Por-GNSs (d), and Fe-Phc-GNSs (f), Figure S10: Variation of resistance of devices based on pristine graphene as a function of different voltage, Figure S11: Statistical measurements of the resistance of GNSs before and after functionalization, Figure S12: The response of GNS sensor and functionalized GNS sensors to NO2 gas under various concentrations ranging from 0.4 ppm to 2 ppm.
