# Supplementary material for: Solution-Processed Functionalized Graphene Film Prepared by Vacuum Filtration for Flexible NO2 Sensors
Source: Sensors (Basel). 2023 Feb 7;23(4):1831. doi: 10.3390/s23041831 (PMC9965048; doi:10.3390/s23041831)
Supplement: Supplementary file 1 [file sensors-23-01831-s001.zip › sensors-2185532-supplementary.pdf]

Solution-Processed Functionalized Graphene Film Prepared by Vacuum Filtration for Flexible NO_2 Sensors

Mbaye Dieng [1,2], Siva Sankar [3], Pingping Ni [1,2], Ileana Florea [2], Pedro Alpuim [3,4], Andrea Capasso [3], Abderrahim Yassar [2] and Fatima Zahra Bouanis [1,2,*]

[1] COSYS-IMSE, Univ. Gustave Eiffel, F-77454 Marne-la-Vallée, France
[2] Laboratory of Physics of Interfaces and Thin Films, UMR 7647 CNRS/Ecole Polytechnique, IP Paris, 91128 Palaiseau, France
[3] International Iberian Nanotechnology Laboratory, 4715-330 Braga, Portugal
[4] Center of Physics, University of Minho, Campus de Gualtar, 4710-057 Braga, Portugal
* Correspondence: fatima.bouanis@univ-eiffel.fr

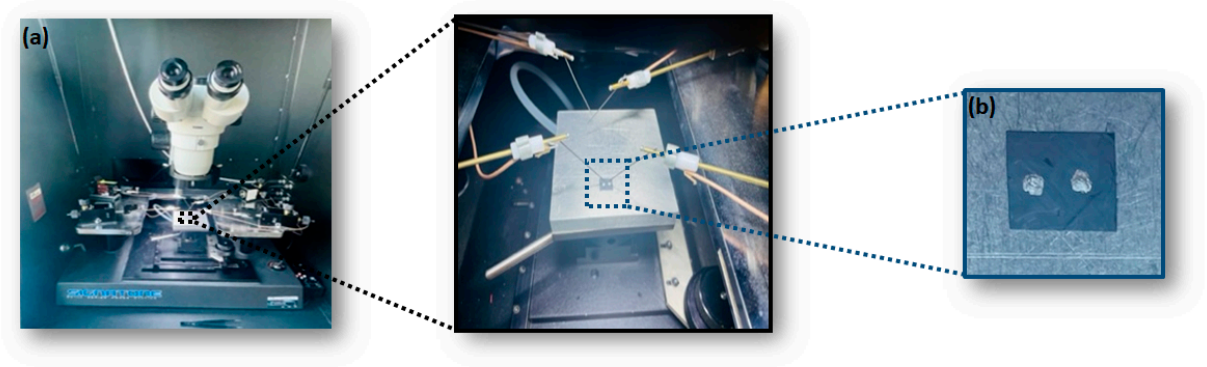

Figure S1. Schematic view of (a) measurement setup for resistance, (b) GNSs sample with silver past.

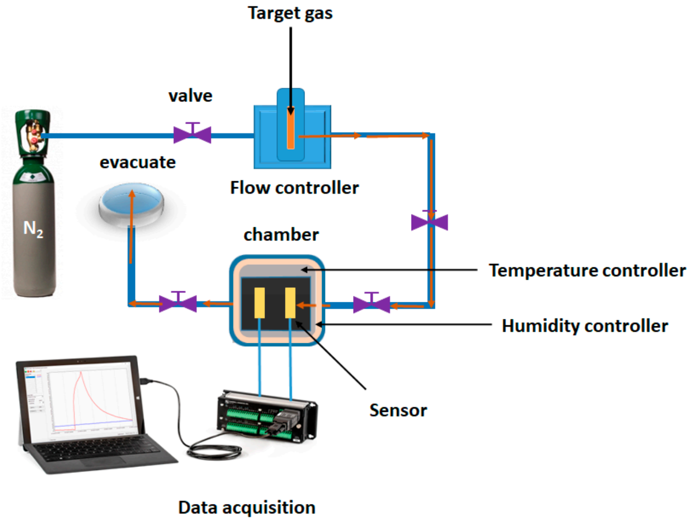

Figure S2. Schematic view of setup used for gas sensing measurement.

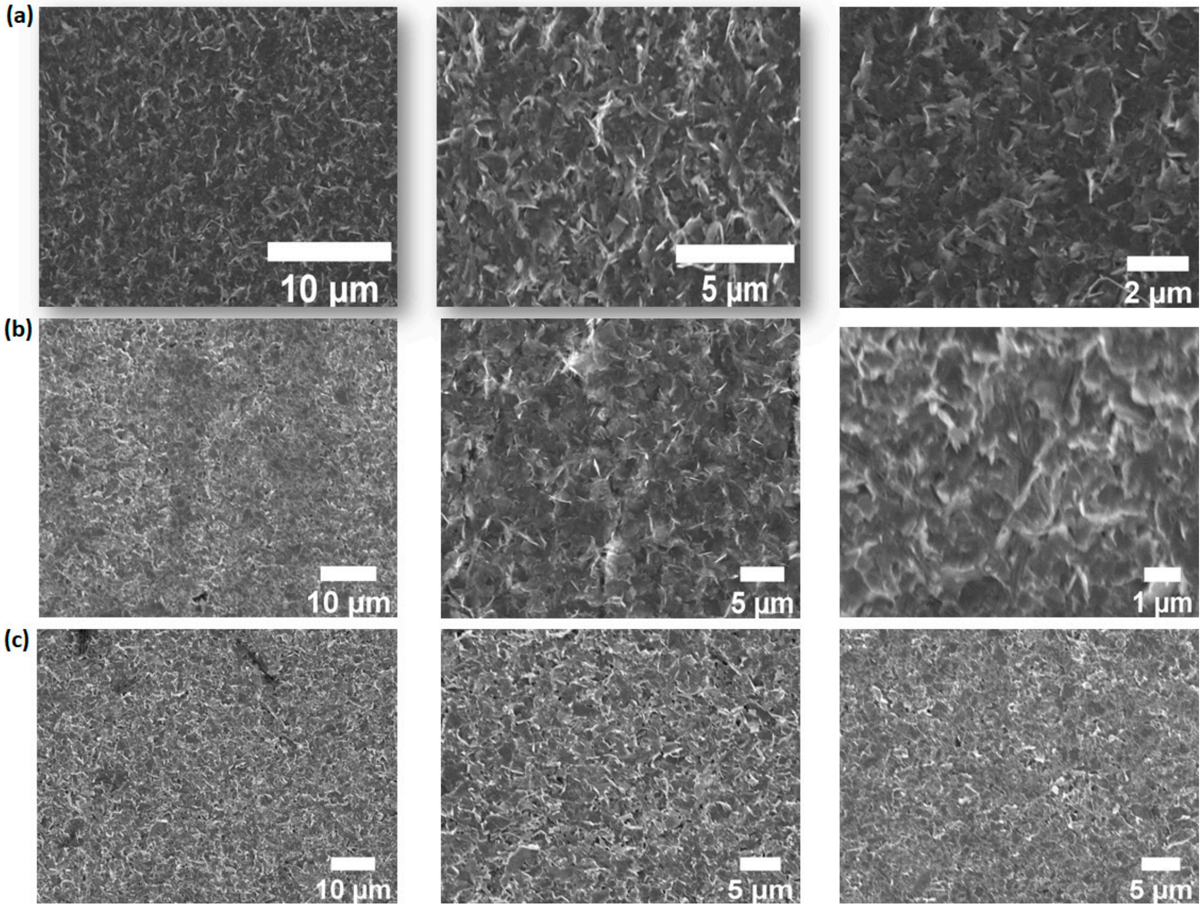

Figure S3. Scanning electron microscopy (SEM) images of (a) GNSs prepared by liquid phase exfoliation and filtered on PVDF membrane, (b) GNSs functionalized with Co-Por and (c) GNSs functionalized with Fe-Phc.

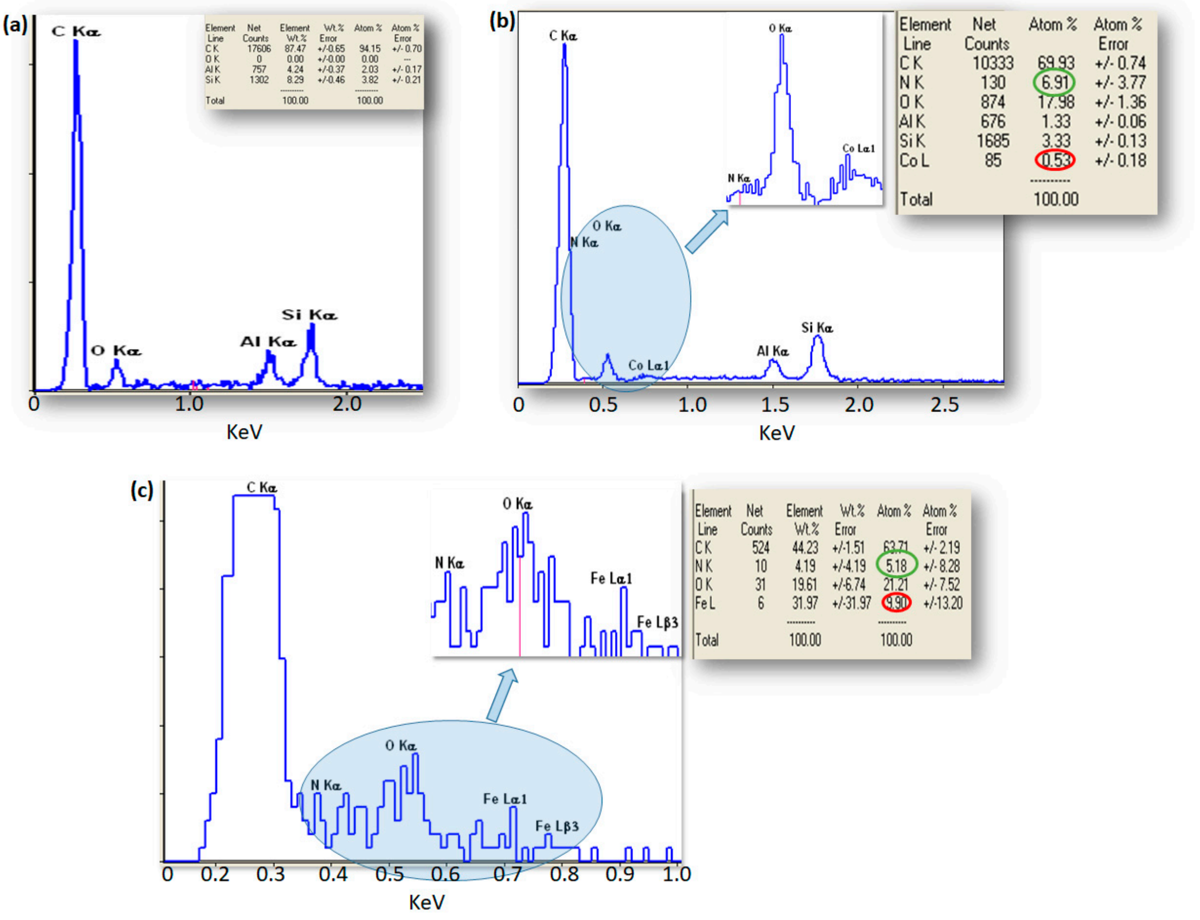

Figure S4. Energy-dispersive X-ray analysis (EDS) spectra of GNSs (a) and functionalized GNSs with Co-Por (b) and Fe-Phc (c).

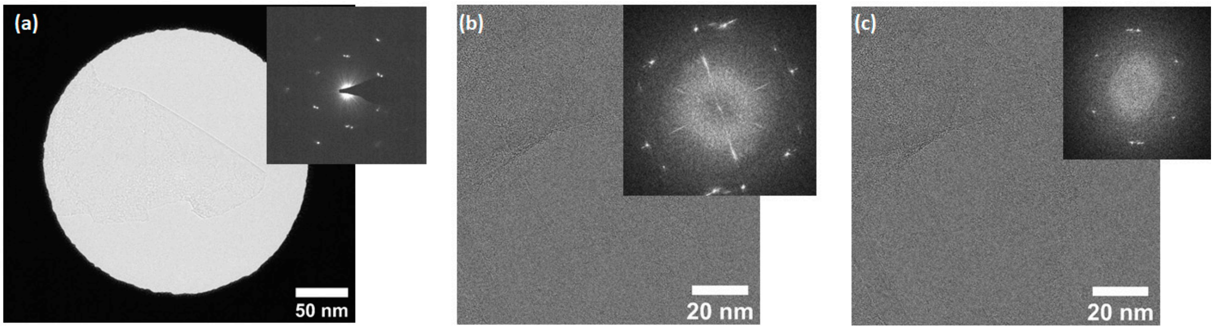

Figure S5. TEM image of GNSs obtained from different areas and its corresponding electron diffraction pattern in inset.

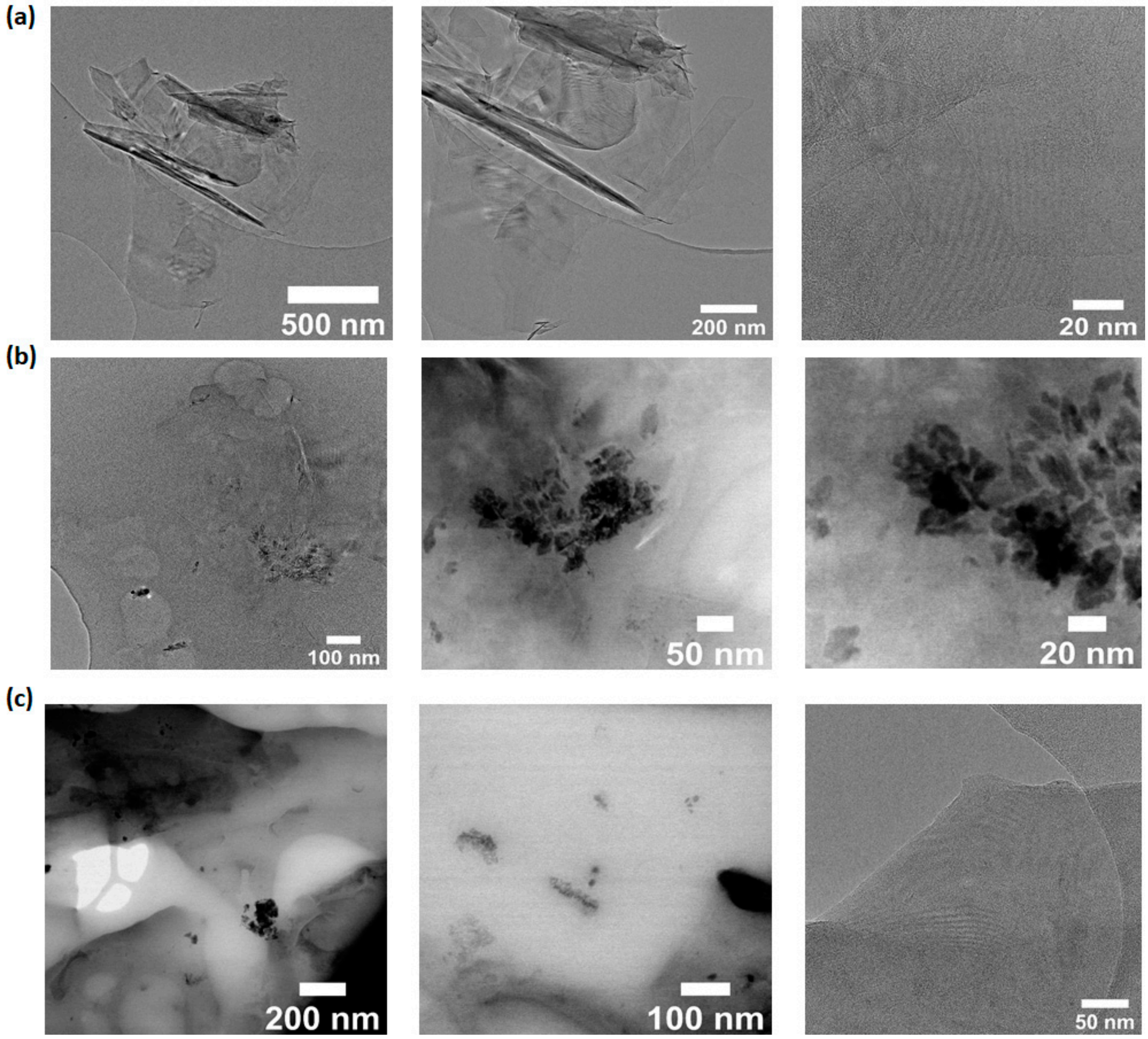

Figure S6. HRTEM and STEM-BF micrographs showing GNSs film (a) obtained from different areas on the surface and functionalized GNSs with Co-Por (b) and Fe-Phc (c).

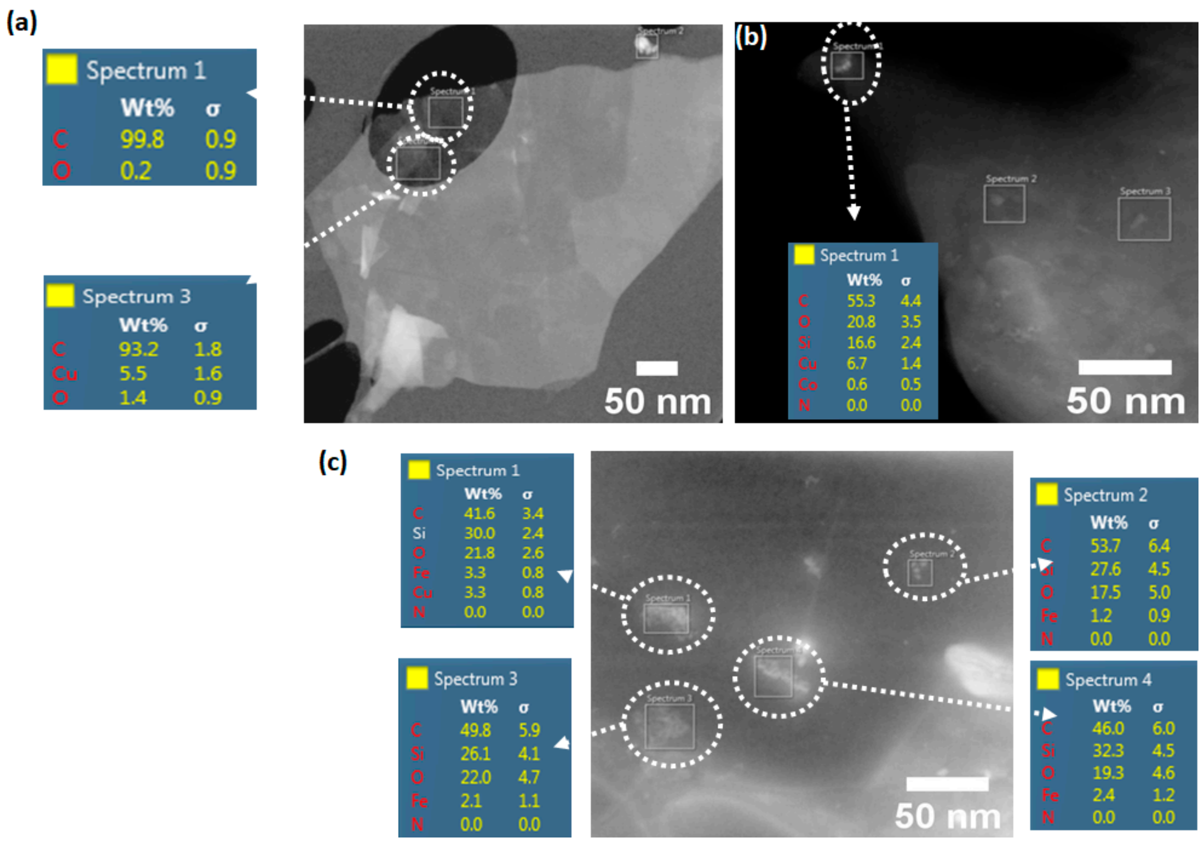

Figure S7. STEM-HAADF-EDS chemical mapping recorded on a larger area of GNSs (a) and functionalized GNSs with Co-Por (b) and Fe-Phc (c) giving and indication of the elemental percentage on each analysed area.

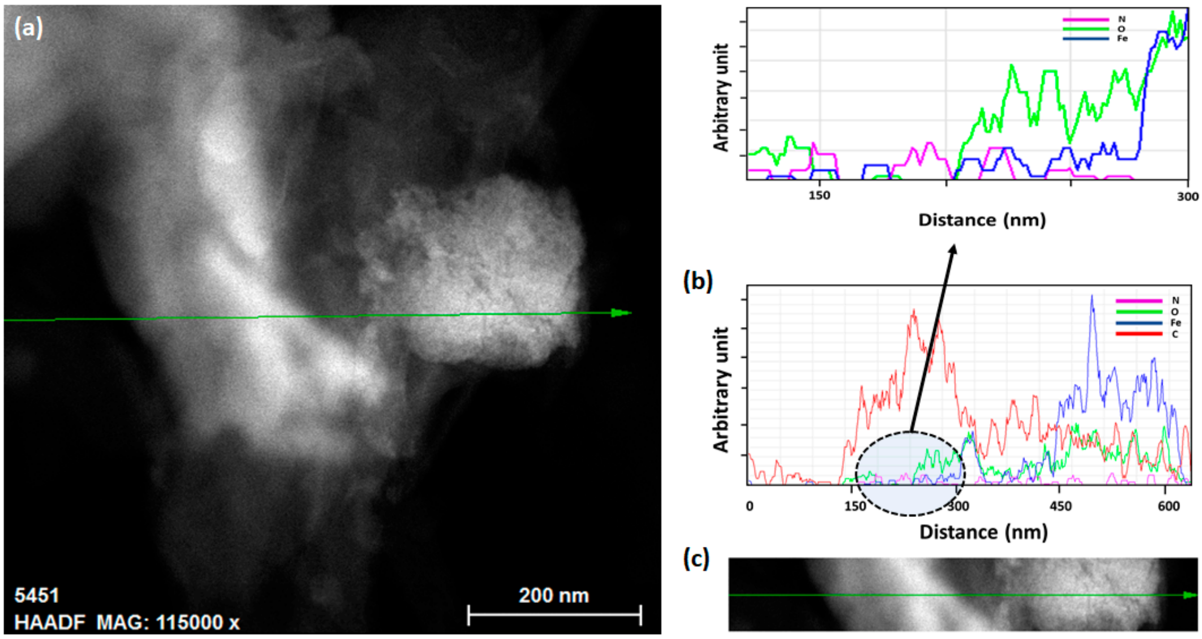

Figure S8. STEM-HAADF-EDS line scan analyses of Fe-Phc functionalized GNSs: (a) STEM-HAADF micrograph on the chosen area where a line scan along the direction indicated by the yellow arrow was performed. (b) Corresponding STEM-EDS line scan profile showing the variation of the Nitrogen (in pink), Iron (in bleu), Carbon (in red) and oxygen (in green) signal recorded along the scanning direction indicated by the green arrow in (c). (c) Zoom of the analyzed area indicated by the yellow arrow in (a).

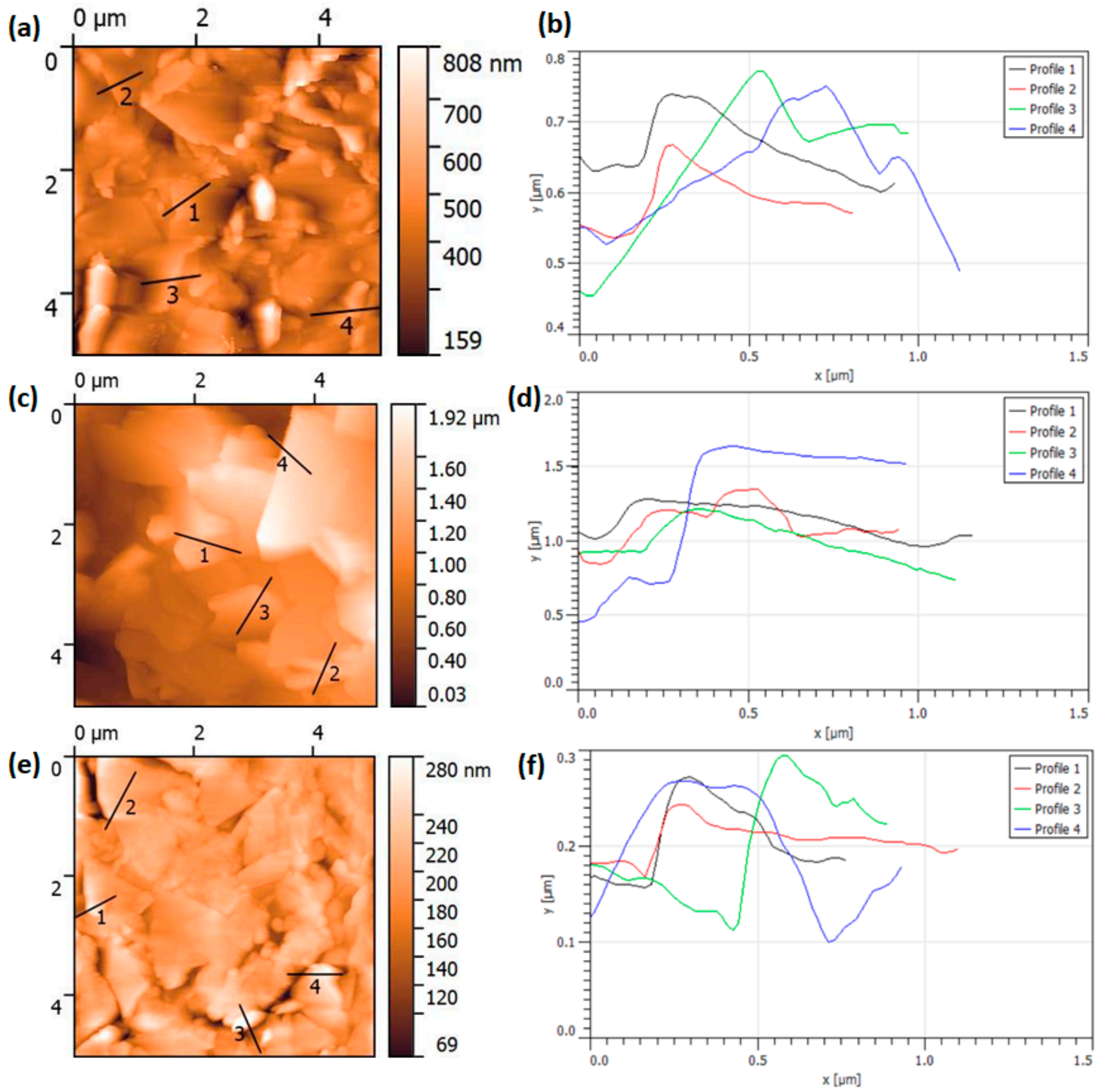

Figure S9. AFM image of GNSs (a), Co-Por-GNSs (c) and Fe-Phc-GNSs (e). Height profile of the black line in image of GNSs (b), Co-Por-GNSs (d) and Fe-Phc-GNSs (f).

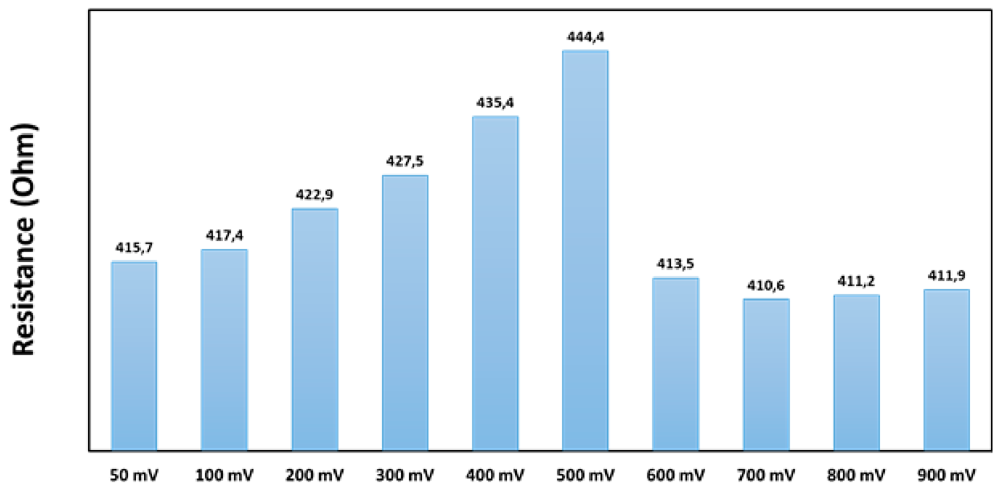

Figure S10. Variation of resistance of devices based on pristine graphene as a function of different voltage.

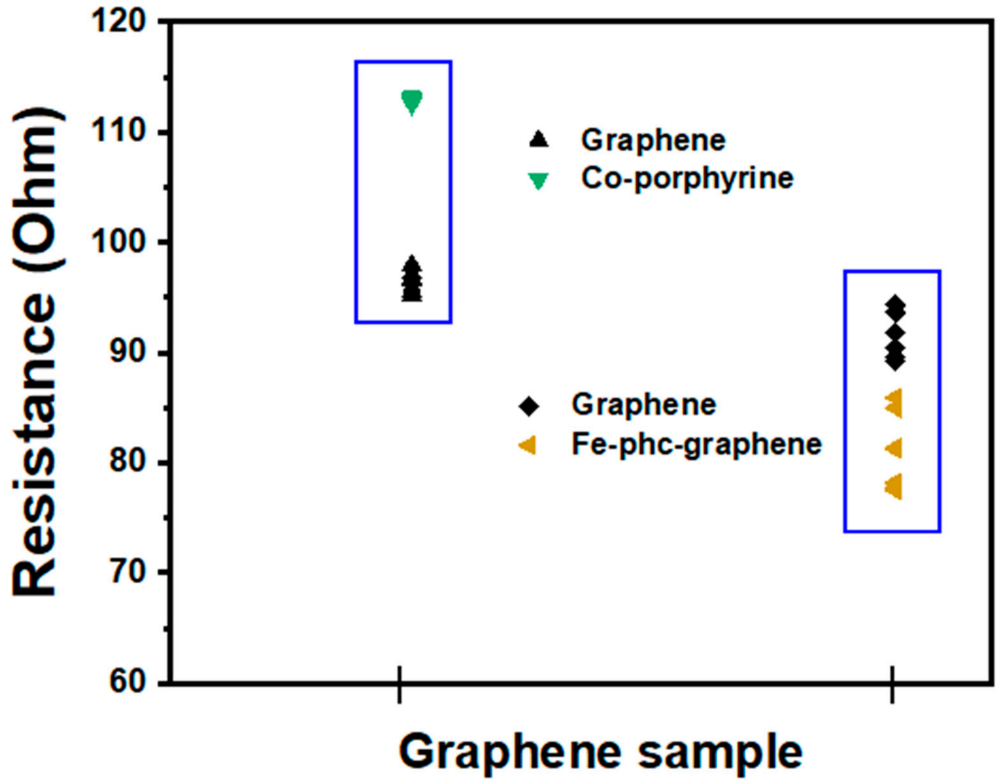

Figure S11. Statistical measurements of the resistance of GNSs before and after functionalization.

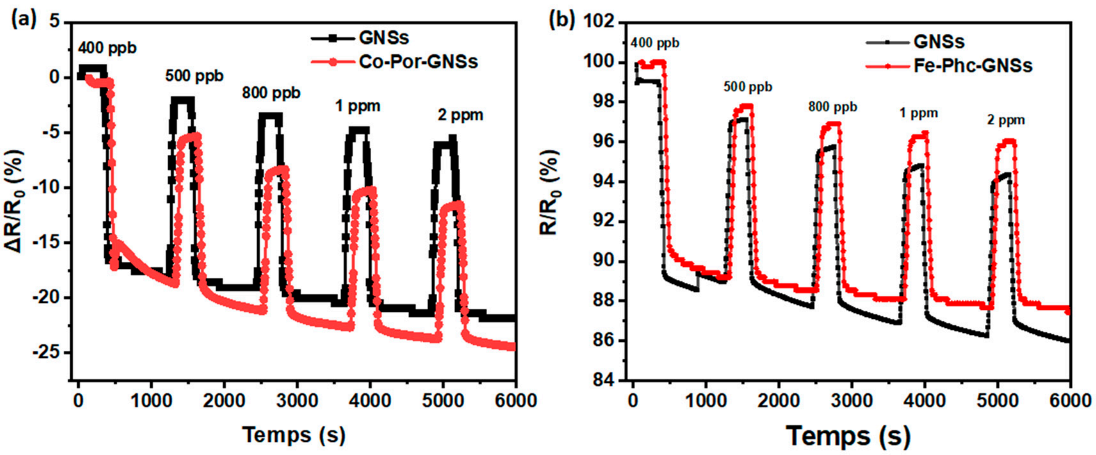

Figure S12. The response of GNSs sensor and Functionalized GNSs sensors to NO_2 gas under various concentrations ranging from 0.4ppm to 2ppm.
